# A Review of Electrically Driven Soft Actuators for Soft Robotics

**DOI:** 10.3390/mi13111881

**Published:** 2022-11-01

**Authors:** Zhaoqi Ma, Dan Sameoto

**Affiliations:** 1 Faculty of Electrical and Computer Engineering, University of Alberta, Edmonton, AB T6G 2R3, Canada; 2 Faculty of Mechanical Engineering, University of Alberta, Edmonton, AB T6G 2R3, Canada

**Keywords:** soft actuators, soft pumps, soft robotics, electrically driven actuators

## Abstract

In recent years, the field of soft robotics has gained much attention by virtue of its aptness to work in certain environments unsuitable for traditional rigid robotics. Along with the uprising field of soft robotics is the increased attention to soft actuators which provide soft machines the ability to move, manipulate, and deform actively. This article provides a focused review of various high-performance and novel electrically driven soft actuators due to their fast response, controllability, softness, and compactness. Furthermore, this review aims to act as a reference guide for building electrically driven soft machines. The focus of this paper lies on the actuation principle of each type of actuator, comprehensive performance comparison across different actuators, and up-to-date applications of each actuator. The range of actuators includes electro-static soft actuators, electro-thermal soft actuators, and electrically driven soft pumps.

## 1. Background

**Figure 1:**
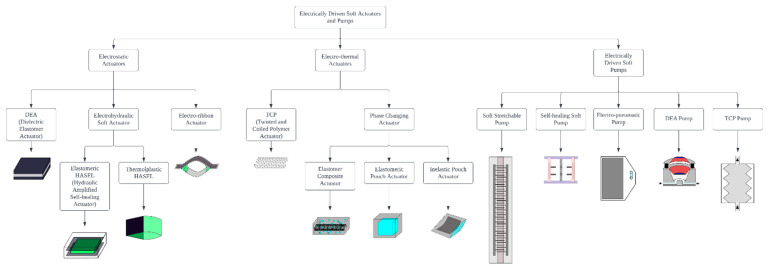
Figure showing different types of actuators being discussed in this review article.

Soft robotics has been around as a field for many decades, starting with McKibben actuators, but exploding in academic popularity in the last 12 years. For most of this time, the vast majority of soft robotics has been powered by some type of pneumatic actuation, i.e., positive or negative pressurized gas (primarily air) [1,2]. This actuation mechanism is conceptually simple, relatively safe, accessible to researchers around the world, and has been the subject of several review articles summarizing the field. However, as soft robotics scale down in size the resistance to the flow of gas through tubes becomes very substantial, and efficiencies drop. The bandwidth of response times for pneumatically driven soft actuators can also be relatively slow (seconds to minutes) depending on the relative size of input tubing and the size of the actuator chambers. In contrast, electrical actuation for traditional robotics has been well established, but within the soft robotics field has been relatively niche because the equivalent of an electromagnetic motor is challenging with soft material. Simple reconstruction of a traditional electromagnetic motor using soft materials would encounter problems such as the hardness of copper coil or higher resistivity of liquid metal such as eGaIn. Traditional motors have rather complicated structures, replacing the traditional rigid structure using a soft one would also be difficult. While notable efforts had been made to implement soft electromagnetic actuators using magnetic powder [3], liquid metal [4], or coated coper wire [5], the designs exhibited different drawbacks such as low force output and efficiency, and moreover they do not actuate in a rotatory fashion like traditional motors. Alternatives are being developed however at a rapid pace, because if an equivalent system for simple manufacturing, high efficacy, and compatibility with existing electrical infrastructure could be developed, then soft robotics could be expanded to a much larger array of industrial, commercial, and household applications. This review therefore will focus on the majority of soft material compatible electrical actuation mechanisms reported in the literature. Figure 1 shows the categorization of the types of actuators being reviewed. For each type of actuator, we have included the basic operating mechanism as well as exemplary demonstrations of applications from the literature. This review does not cover IPMC (ionic polymer-metal composite) and shape memory alloy/polymer, a detailed review of them, along with some topics covered in this review, was composed by Ahn et al. [6].

## 2. Dielectric Elastomer Actuator

**Figure 2:**
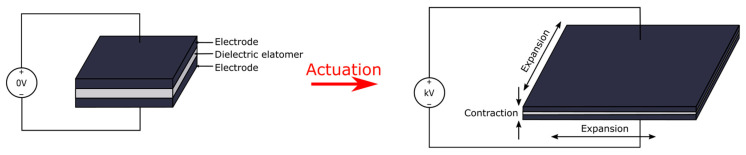
The actuation scheme of a typical sandwich-structured DEA. Upon the application of a high voltage (typically in the kilovolts range), the electrostatic force created between the top and bottom electrodes will squeeze the actuator, thus creating areal expansion and linear contraction in the third dimension (thickness in this case).

Dielectric elastomer actuators (DEAs) are a class of soft and flexible actuator that deform under high applied voltage (usually in the kilovolt range) and returns to their original shape after the voltage is removed. It is one of the most studied and commonly used actuators in soft robotics and artificial muscles due to the advantage of directly converting electrical energy to mechanical energy and the linear actuation that closely mimics human muscle [7]. DEAs are often made of 3 elastomer layers that resemble a sandwich structure as shown in Figure 2, with the top and bottom layers being electrodes often made of carbon grease, graphite, carbon nanotubes, or carbon black [8,9,10,11,12], and the middle layer being a dielectric elastomer often made by silicone, acrylic, or polyurethane (PU). Upon activation using high voltage, the generated Maxwell stress between the two electrodes will deform the actuator by compressing the dielectric elastomer, and when the electrical potential is removed, the elastic force of the elastomer will restore the actuator [13]. DEAs have been shown to achieve great performances in strain, bandwidth, and energy efficiency. Linear stains over 200% [14,15], areal strains over 300% [16], and bandwidths over 500 Hz were reported [17,18]. However, most of these outstanding performances are demonstrated under pre-stretched conditions, meaning that a soft DEA membrane is being stretched and fixed to a rigid frame before the application of voltage. In most cases, DEAs without pre-stretching show strains below 100% and worse performance in other parameters. A major drawback of DEAs is the requirement of high voltage amplifiers to operate, making the safety and durability of the actuator a much larger concern than pneumatic-based soft actuators.

A DEA directly converts electrical energy into mechanical energy upon an applied voltage. The degree of actuation motion can be controlled by a microcontroller unit (MCU), primarily in open loop control but occasionally using capacitive feedback. Furthermore, the simple and solid structure of DEA has made it resistant to external damage like falls and crushing, pressure-resistant, and capable of reacting to high-frequency voltages at speeds limited by the mechanical response of the rubber. These advantages have made DEAs an excellent choice for soft locomotive soft robots [17,18,19]. By leveraging the pressure-resistance properties of DEA, Li et al. recently presented a swimming soft robot that mimicked the deep-water snailfish and successfully swam in the Mariana trench (10,900 m underwater) for 45 min (on battery) without succumbing to the enormous water pressure [19]. The fins of the robot were actuated by two specialized styrene-*b*-butyl acrylate-*b*-styrene (SBAS) DEAs pre-stretched onto two rigid frames that connected the fins to the body. The specially designed geometry of the rigid frames [20] would cause the fins to flap upon actuation. The choice for SBAS was due to its low glass-transition temperature, which ensured the functionality of the actuator under the extreme conditions of 110 MPa water pressure and 2.7 °C temperature. As a result, the actuator could achieve 7% strain at 5 °C and 110 MPa, and the 22 cm long robot achieved a free-swimming speed of 2.76 cm s^−1^ in a 110 MPa pressurized chamber when activated using a 7 kV 1 Hz signal. The high actuation frequency of DEAs has been proven useful for building insect mimetic soft robots. Chen et al. had proposed a controllable soft aviator that was built by a multilayered cylindrical DEA with four flapping wings [17]. The actuation peaked at 15% strain when operated at a resonance frequency of 500 Hz and was responsive to 1000 Hz input frequency. Low voltage actuation below 500 V was achieved by Ji et al. by reducing the thickness of each DEA layer to the few μm range [18]. The group presented a low-voltage stacked DEA (LVSDEA) that was built by 3 layers of circular PDMS elastomer (3 mm radius and 6 um thickness) with 4 layers of soft and stretchable single-walled carbon nanotube (SWCNT) electrodes. The group also built a soft crawler called DEAnsect by attaching 3 LVSDEAs with 3 directional frictional feet. Upon activation using 450 V, 450 Hz signal, the 40 mm long DEAnsect crawled at 30 mm/s (0.85 BL/s) under tethered conditions, and when the frequency was tuned to 390 Hz, a speed of 12 mm/s was achieved under an untethered condition in which the controller and power units, weighing 780 mg, were attached to the 190 mg body.

Other than locomotive robots, DEA has also been used for force generation in other applications. Scaling up of output force and strain by stacking of DEA was shown by Duduta et al. [9]. The group stacked 1170 layers of disk form factor DEAs to create a 6.3 cm long artificial muscle that weighed 20 g and lifted 1 kg of load by 8 mm upon 3.5 kV applied voltage. Efficient fabrication of the disks was done by using UV-curable acrylic dielectric elastomers with low density (15 μL cm^−3^) CNT ink electrodes. The stacked actuator reached a peak strain of 24% and energy density of 19.8 J/kg, both of which exceeded the typical values of a mammal muscle [21]. Nevertheless, low actuation bandwidth of 0.1 Hz at 3 kV was shown for full actuation.

In terms of fabrication, the simplicity of structure allowed 3D printing of dielectric elastomer fiber (DEF) to be possible [22,23]. While the majority of DEAs have been based on 2D electrodes/actuator arrays either built singly or stacked together, recently, Chortos et al. proposed a directly printable DEF in a cylindrical form factor [23]. The “inks” for electrode and dielectric matrix were carefully chosen so that they could flow through the nozzle under an adequate shear stress and return to a solid-like state almost immediately after exiting the nozzle. The group used a mixture of Ecoflex 30 silicone, SE1700 silicone, and PDMS-functionalized fumed silica for the dielectric matrix to balance pre-printing fidelity and post-curing mechanical properties. The electrode was made of a silicone matrix with carbonblack added for electrical functionality. The printed actuator possessed a cylindrical structure with a solid conductive core, a dielectric middle layer, and a conductive outer layer. When voltage was applied, the fibers elongated along the axis direction, and a 5 × 5 bundle actuator reached a maximum of 12% linear strain under 4 Hz resonance frequency.

3D printing of 2D sandwich structured DEA was also reported by Haghiashtiani et al. [24]. The group fabricated a 4-layered bending DEA with a motion-straining layer at the bottom and a typical 3-layered DEA attached on top. The DEA portion was built with ionic hydrogel electrodes and silicon dielectric elastomer. Like Chortos et al.’s design, this DEA was also fabricated using a direct-ink-writing process along with carefully prepared extrusion materials. However, instead of using multi-material extrusion to print the DEA at once, this design used different materials in different layers and cured each layer before the application of the next. UV curing was applied to the ionic hydrogel to bond it with the hydrophobic silicon surface. The finished design showed decent tip displacement of 9.78 mm, which was over 6 times its thickness and almost 1/3 of its length, under 5.44 kV applied voltage.

An early study conducted by Kofod et al. utilized the morphology during relaxation from a pre-stretched state to accomplish the self-assembly of DEAs [25]. The group demonstrated a triangular self-assembled DEA gripper by using that strategy. DEAs have been applied to many other applications including haptic feedback [26] for gaming systems, soft microvalves [27], varifocal lenses, active vibration cancellation, tactile displays, and loudspeakers [28]. Small-scale moving parts can also be directly actuated by DEA in the future.

With respect to the actuator itself, there are two major research directions within the literature: optimization of maximum strain for a given voltage and self-healing of the actuators if a dielectric breakdown occurs. A straightforward method for improving maximum strain is using softer materials (lower Young’s modulus) for dielectric elastomers so that higher strain can be achieved under the same stress, as suggested by Yu and Skov [29]. Soft materials such as VHB acrylic and HS-3 silicone have been shown by Perlrine et al. to raise a DEA’s areal strains over 100% [30]. Another method to improve strain is to increase dielectric strength and electric permittivity of the DEA because higher dielectric strength permits higher voltage to be applied before a dielectric breakdown, and higher permittivity boosts the Maxwell stress under the same applied field. A common measure to elevate dielectric strength is pre-stretching the actuator on a rigid frame to reduce spatial defects that cause early breakdown [31]. A significant drawback of this method is the requirement of a rigid frame which is undesirable for a fully soft machine. Thus, enhancing dielectric strength by synthesizing new materials has become a hot research field recently. For instance, diluting silicone with silicone oil has been reported to greatly increase the dielectric strength while reducing the Young’s modulus of the elastomer by Yu and Skov [29]. Embedding CCTO (calcium copper titanate which has a high dielectric constant) into PDMS (polydimethylsiloxane) [32] and synthesizing organic acrylic resin elastomers using a mixture of PTSA (*p*-toluene sulfonic acid) with PANI (polyaniline) [33,34] have also been presented as synthesized elastomers with excellent dielectric constants and strengths. Dielectric strength can also be improved by using carefully designed carbon nanotube electrodes as reported by Duduta et al. [9]. The group suggested that dielectric strength is inversely related to the areal density of conductive carbon nanotube ink, hence claiming that using a thin layer of evenly distributed carbon nanotube ink as electrodes could considerably enhance dielectric strength.

Self-healing is a method to ameliorate the limited lifetime due to dielectric breakdowns and subsequent introduction of short circuits via damaged areas. The hole burnt during breakdown will be filled with gases that are of lower dielectric strengths, making the hole a defective spot that is prone to experience breakdown again. As a result, the defective spot significantly reduces the maximum operating voltage and performance of the whole actuator. To solve the problem, several “self-healing” DEAs have been proposed. Hunt et al. used a combination of open-cell silicone sponge with silicone oil to achieve self-healing via the autonomous refill of burnt holes with silicone oil after a breakdown occurs [35]. Duan et al. proposed a DEA made of self-healing PDMS-PANI elastomer that can self-heal up to 35.14% of pristine strain-stress property within an hour, and 97.98% within 4 h from autonomous adhesion under room temperature conditions [36]. La and Lau showed how encapsulating an acrylic DEA within silicone gel can prevent early breakdown by eliminating contact between electrode and oxygen to suppress partial discharges [37]. The encapsulation is also found to help confine the expansion of holes by limiting the combustion during breakdown. Not only are dielectric elastomers affected by breakdown, but electrodes can also come damaged. To address this issue, self-clearing electrodes have been presented. As an example, Peng et al. proposed a water-based polyurethane + single-walled carbon nanotube bilayer compliant electrode that exhibited self-clearing characteristics [38].

A future research direction of DEA can be oriented toward developing an ideal DEA that is made of ultra-soft elastomers that exhibit superior dielectric properties based on fast self-healing polymers like the ultrafast healing PDMS [39] and peptidoglycan-inspired bio-friendly elastomer [40]. Another direction can be aimed at low-voltage actuation of larger DEAs (thickness in the mm range). Even though some research groups have developed compact voltage amplifiers that can be integrated into soft robots for untethered actuation [18,19], high voltage actuation still possesses safety and cost problems. Low voltage DEA, likes the ones presented by Ji et al. [17] is a work-around to the voltage-amplifier and safety issues. More extreme than Ji et al.’s design is Poulin’s DEA design, which was only 3 um in thickness [41]. By doing so, the group managed to reach an activation strain of 7.5% under a low voltage of 245 V. Therefore, mass fabrication of extremely thin DEAs and stacking them together could potentially build a novel DEA that exhibits both low voltage actuation and large strain, which may become a great improvement to the field.

## 3. Electrohydraulic Soft Actuators

Electrohydraulic soft actuators are soft actuators that utilize electrostatic force for actuation with the addition of dielectric liquid contained within a solid pouch rather than elastomers as the insulating layer. The most representative electrohydraulic actuators are the HASEL (hydraulic amplified self-healing electrostatic) actuators first reported by Kellaris et al. and Acome et al. [42,43]. The HASEL actuators can be separated into two categories: elastomeric HASEL and thermoplastic HASEL, with the former having an elastomeric shell and the latter having a relatively inextensible but thin and flexible thermoplastic shell. Elastomeric HASELs can be viewed as softer DEAs with the capability to self-heal, and therefore have higher theoretical strains and longer lifetimes. Thermoplastic HASELs, on the other hand, are quite different from the typical DEA form factor. Thermoplastic HASELs are pouch actuators that exhibit the merits of lightweight, high theoretical specific energy, cheap fabrication, and the ability to be easily fabricated into complex shapes.

**Figure 3:**
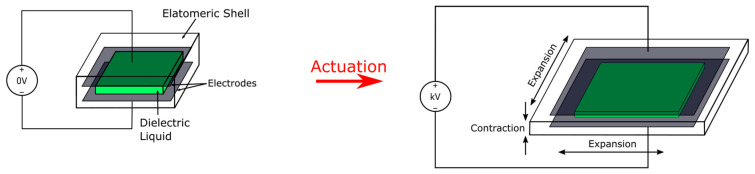
The actuation scheme of a typical elastomeric HASEL. Similar to a sandwich-structured DEA, an elastomeric HASEL requires high voltage (kV range) to actuate, and the actuation scheme is also planar expansion + linear contraction. The dielectric liquid core has a much lower Young’s modulus than the elastomer core of a DEA, thus making a similarly shaped elastomeric HASEL have higher actuation strain under the same actuation conditions (i.e., applied voltage and pre-straining).

The elastomeric HASEL, reported by Acome et al. [42], can be viewed as a DEA with a liquid dielectric filling inside a dielectric elastomer shell as shown in Figure 3. Hence, elastomeric HASELs also use Maxwell stress to actuate. A non-linear actuation pattern due to pull-in instability was reported. The lower effective Young’s modulus of dielectric liquid filled elastomer enables HASELs to perform larger strain than a similar shaped DEA for a given voltage, while the restorative force of the elastomeric shell allows a comparable bandwidth to DEAs. For comparison, a circular HASEL was shown to outperform a DEA of similar shape and material by producing 4× the DEA’s areal strain at 11 kV applied voltage. The study also showed a rectangular shaped planar HASEL reaching a maximum linear strain of 107% and 79% under 205 g loading condition with and without mechanical resonance respectively. The use of dielectric fluid furthermore provided the ability to self-heal by filling in defective spots after dielectric breakdowns.

Akin to DEA, elastomeric HASEL can also be modeled as a capacitor, therefore providing the ability for self-sensing and closed loop controlling by monitoring the capacitance between two electrodes [42,44,45]. Furthermore, 3D printing of elastomeric HASEL has also been developed by O’Neil et al. to produce a complex tentacle structure by using the continuous liquid interface production method [46]. In the study, photocured silicone urethane was used for an elastomeric body, and electrodes made of hydrogel and silver paint were injected into printed body layers. Despite all the merits, elastomeric HASEL has not been extensively studied up to date, and applications have remained at a preliminary level. This may be caused by its similar with DEA, relatively complicated fabrication, and recentness. Future studies can be focused on replacing existing DEA designs with elastomeric HASEL to achieve better strain performance.

**Figure 4:**
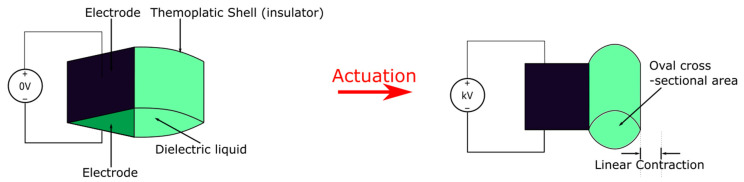
The actuation scheme of a single thermoplastic HASEL pouch. Upon application of a high voltage (kV range), an electrostatic force will form between the two electrodes (black portion) and get further amplified through the dielectric liquid. This force causes the two electrodes to pull together and squeeze the liquid out of the region covered by the electrodes. This will cause the uncovered pouch to form an oval-shaped cross-sectional area and the shape-change ultimately creates a linear contraction in the length direction.

A more widely reported version of HASELs, the thermoplastic HASEL [43,47,48,49], is a pouch actuator that utilizes electrostatic zipping instead of electrostatic pulling for actuation as shown in Figure 4. Thermoplastic HASEL is typically made of thermally bonded inelastic films that form pouches, and each pouch is filled with a dielectric liquid. A portion of the pouch is bonded with aligning electrodes on two sides, and upon actuation via high voltage (kV), this active portion will gradually zip together due to electrostatic force being amplified through the dielectric liquid. The zipping will displace dielectric liquid from the active region to the inactive region (the portion of the pouch without electrodes) and bulge that region, causing an overall contraction in the length of the actuator. Thermoplastic HASELs also exhibit the ability to self-heal over 50 dielectric breakdowns from the dielectric liquid [43], self-sense position from monitoring capacitance, and hence closed-loop control from self-sensing is possible [50]. A major benefit of the thermoplastic HASEL design is its’ easy and relatively cheap prototyping, which makes future developments highly efficient. Typically, a thermoplastic HASEL can be fabricated with four simple steps: (1) thermal bonding of thermoplastic sheets, (2) fluid injection, (3) sealing, and (4) adhering electrodes. Intricate shapes of the pouches can be fabricated using modified extrusion heads of 3D printers [51]. In terms of cost, Kellaris et al. estimated a material cost of $0.1 per hydrogel-electrode Peano-HASEL actuator [43]. This feature also makes thermoplastic HASELs very suitable for mass-production applications and single use bio-medical applications. Moreover, the special liquid characteristic and simple structure make it very versatile. However, the thin plastic shell makes the actuators much more susceptible to physical damage than solid DEA and the mechanical performances are inferior to top-class DEAs. Healing of the thermoplastic shell and subsequent leaking of dielectric may also be a long-term concern.

The first thermoplastic HASEL actuator was the Peano-HASEL built by Kellaris et al. [43]. The actuator was comprised of three serially connected rectangular bi-axially oriented polypropylene (BOPP) pouches filled with vegetable-based transformer oil (Envirotemp FR3 and Drakeol 7) and attached to ionically conductive hydrogel electrodes. A single actuator exhibited a maximum of 10% strain under 20 g loading condition at 10 kV applied voltage. A stack of 6 actuators in parallel (weighing 30 g) could lift a 500 g load over a strain of 4.6% at 8 kV. The frequency-dependent amplitude response of the actuator was nearly constant until 25 Hz where the actuation strain dropped below 90% of maximum static strain, and the actuator remained responsive to 50 Hz actuation signal.

To improve on the maximum theoretical strain of a Peano-HASEL, Wang et al. proposed a high-strain HASEL (HS-HASEL) that had two pairs of electrodes located at the two ends of the horizontal direction to perform zipping motion that was orthogonal to the vertical direction of contraction [49]. The maximum strain of HS-PEANO was 24%, well surpassing the 10% of Peano-HASEL. As an application, the study presented an artificial circular muscle by wrapping 12 units of HS-HASEL around a silicone tube. Upon activation, the swelling of actuators would cause the contraction of the silicone tube. The circular muscle was demonstrated as a pump that could produce a maximum air pressure of 2.73 kPa, a peak flow rate of 2.3 L/min, and an average rate of 1.47 L/min under 4 Hz, 10 kV sinusoidal driving signal. Intricate actuator designs fabricated through heated extrusion heads were demonstrated by Mitchell et al. [51]. The study presented a curling artificial scorpion tail that achieved a linear speed of 1.26 m/s, an angular velocity of 1987°/s, and a blocked tail force of 550 mN. The group used the fabrication technique to enhance the design of a donut HASEL, and the new design achieved 118% strain under 100 g loading at 12 kV when three donut actuators were stacked. A high blocking force of 60 N was reported after which the failure mode was determined to be a rupture of the heat-sealed bond between thermoplastic layers. If a restorative force was provided by a spring, the stack exhibited a flat frequency response at 25% strain with a bandwidth of 126 Hz.

By using the expansion of the inactive portion of HASELs, Kellaris et al. built a spider-inspired electrohydraulic soft actuated joint (SES) that performed bending (rotational) motion instead of linear contraction [52]. In the design, two stiff plates were connected via an elastomer hinge, and a thermoplastic HASEL is connected two the two plates. When activated, two stiff plates would bend to a designated angle controlled by the applied voltage, and the hinge would expand. Once voltage was removed, the hinge would restore the original state of the SES. The SES presented a maximum rotation angle of 70 degrees, a blocking torque of 70 mNm, a specific power of 230 W kg^−1,^ and bandwidth of 24 Hz. In the study, a jumping robot powered by a single SES joint was demonstrated to jump up 5.5 cm (10 times its height) upon a square voltage, and a 3-finger soft gripper with each finger powered by two SES joints could grab up a 270 g ceramic mug. Furthermore, Purnendu et al. extended the concept to origami designs by integrating HASELs into the folded hinges to activate the bending movements of certain parts [53,54].

Two degrees of freedom on the same actuator can be achieved by attaching electrode pairs of different sizes and shapes on the same shell, as shown by Kim and Cha [44,47]. The larger pair displaced more liquid and thus resulted in larger deformation while the smaller pair performed a smaller-scaled actuation. Placing four of such actuators under a circular plate, Kim and Cha were able to create an active table that rolled in a ball in a circular path.

Newer applications of HASELs are being introduced, including indirect actuation, or integration with more complex robotics. For example, Park et al. demonstrated a curling actuator by combining thermoplastic HASEL with a swelling pouch actuator and built a soft gripper with two curling actuators [48]. The gripper could grasp objects such as a Ping Pong ball and a balloon when activated at 10 kV. Thermoplastic HASELs have also been shown as a substitute for traditional motors in the field of prosthetics due to their advantages of silent operation lightweight and linear actuation. Yoder et al. presented a prosthetic finger driven by an array of Peano-HASELs [55]. The HASEL array reached 91% of the maximum motion and 10% of the maximum fingertip force achieved by using a DC motor actuator, while consuming 8.7 times less energy, operating 10.6 times faster, and having 11.1 times higher bandwidth. Shape morphing designs, such as tangible animation inside a book and physical character animation, can be activated by thermoplastic HASEL as shown by Purnendu et al. [54]. The group demonstrated the concept of Electriflow, or in other words the use of electro hydraulic actuators as building blocks for shape-changing interfaces. Software for assisting the design of electrohydraulic actuator prototypes is also included in the study. Other applications include a varifocal lens based on an electrohydraulic actuator made by three layers of circular transparent PDMS and a pair of annular unstretchable electrodes [56] and a haptic feedback actuator made of a similar circular structure [57].

In the future, research directions can be aimed either to optimize thermoplastic HASEL performances or at expanding its applications. Force optimization is possible by increasing the pouch density of an array by miniaturizing each pouch and fitting more pouches into the same area. As suggested by Kellaris et al. [58], pouch length does not affect the force output of each pouch, thus fitting more pouches within the same length can exert multiple times the force while performing a similar strain. Models predicted a specific energy over 10,000 J/kg if pouches can be reduced to sub-mm sizes. Fabrication using thin metalized films or electroactive polymer films with lithography or etching techniques may enhance the miniaturization of design. The lifetime of thermoplastic HASELs can be augmented by applying better sealing techniques than thermal-bonding because heat-sealing the shell could damage thermoplastic and thus lead to premature breakdown [44]. Another possible improvement is to use self-healing dielectric fluid that solidifies upon contact with air since dielectric breakdown will burn a hole in the thermoplastic shell and hence cause leakage of dielectric liquid if the hole is left untreated. In terms of application, thermoplastic HASEL can be applied to locomotive robots and has great potential for biomedical applications and large-scale production.

## 4. Electro-Ribbon Actuator

**Figure 5:**
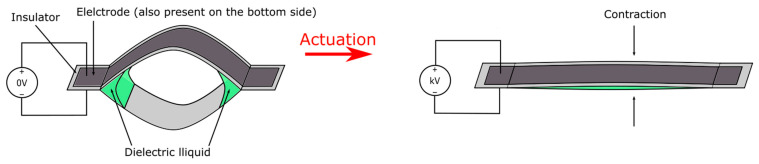
The actuation scheme of an electro-ribbon actuator. Upon applied voltage (in the kV) range, a non-uniform electric field (in the length direction) will be formed between the two electrodes with the strongest fields at the two hinges and the weakest field at the center, causing the two tiles to zip together from the two hinges.

The Electro-ribbon actuator, as shown in Figure 5, is another type of electrostatic actuator developed by Taghavi et al. that folds or clamps upon activation [59]. It uses a principle called dielectrophoretic zipping, which means zipping using Maxwell force with the amplification of a bead of dielectric liquid. Compared to thermoplastic HASELs, this type of actuator does not need an encapsulation to hold a considerable volume of liquid and thus is lighter. This actuator only requires a bead of liquid to amplify the whole zipping process, and that bead will not leak out due to the dielectrophoretic effect [59,60] although if excess liquid is applied, then some may be lost in practice. The concept of this design is simple but useful: any fold can be actively zipped closed if electrodes and dielectric liquid are present within the fold hinge. As the fold closes, the liquid is pushed ahead to enhance the force as the sides get closer together. Electro-ribbons have shown a maximum contraction of 99.84%, specific energy of 6.88 J/kg, specific power of 103.51 W/kg, and energy efficiency of 70% [59]. Scaling up the strain and stress can be done by series and parallel connections respectively. However, a bandwidth of 0.125 Hz for full zipping and 10 Hz for partial zipping is lower than DEA and HASEL. The maximum stress of the electro-ribbon is 0.048 MPa, orders lower than Peano-HASEL. In terms of application, the unique dielectrophoretic zipping mechanism makes electro-ribbon the best actuator for active origami-designs because it can be integrated directly into origami folds. For instance, the researchers showed electroactive origami designs of the adaptive gripper, paper spring, locomotion robot, foldable fan, and an origami crane. The simplicity of the working mechanism allows any combination of flexible insulator and conductor, even pencil and paper, to be crafted into an electro-ribbon or electroactive origami.

Self-locking mechanism of electro-ribbon due to pull-in instability was proposed by Taghavi et al. in 2020 [61]. To demonstrate the phenomenon, an actuator was fully zipped under 8 kV, yet the zip persisted even if the voltage was gradually decreased. Unzipping happened when the voltage dropped below 0.4 kV. This phenomenon rendered electro-ribbon a potential alternative to traditional state-transitioning components like solenoid valves. Contraction self-sensing and closed-loop controlling were studied by Bluette et al. [62]. Self-sensing was done through capacitance monitoring and a closed loop control was implemented using a proportional-and-integral (PI) controller. The controller could maneuver the zipping position when a load of known weight was applied. An enhanced closed loop controller, built by Diteesawat et al., enabled electro-ribbon to perform stepping and oscillatory motions [63].

While electro-ribbons exhibit great actuation strain and power efficiency, the actuation speed is not comparable to that of the other electrostatic actuators. Along with other electrostatic actuators, this one also requires high voltage to operate, imposing safety issues for close-to-human applications. Furthermore, the exposed dielectric liquid can flow out of the actuator as shown by the videos provided by Taghavi et al. [59]. This causes the volume of liquid to be reduced over repeated actuations. The exposed liquid can also create challenges on actuator surroundings or potentially become contaminated. This may also create issues for application where active hinges are in contact with a liquid surrounding (i.e., underwater application) or where frequent shocks are anticipated.

Future research directions for this actuator can be oriented toward finding a suitable encapsulation method for the dielectric liquid so that the liquid will stay after a large stroke or impact while the actuation speed and strain are not overly affected. Due to the simple structural design, this actuator can be easily scaled down; therefore, another direction can be integrating this actuator into miniature origami designs in which the use of other types of actuators can be very difficult.

## 5. Electro-Thermal Actuators

### 5.1. Twisted and Coiled Actuator

**Figure 6:**
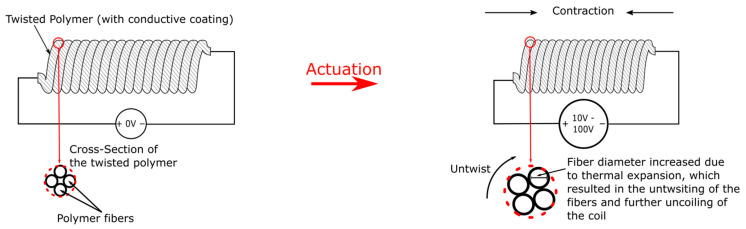
The actuation scheme of a contracting twisted and coiled polymer. The conductive coating of the twisted fibers allows for Joule-heated actuation, and usually around 10–20 V/cm actuation voltage is required for significant contraction, but this is strongly dependent on the chosen polymer and coating material (i.e., resistivity, heat conductivity, etc.). Heating will cause the fiber diameter to increase, which induces an untwisting motion of the fiber, and this will ultimately cause the uncoiling of the coil, creating a contracting motion.

As stated above, electrostatic actuators, despite all its merits, mostly require a high voltage to actuate. This proposes a safety issue for many applications, especially the ones where human-interaction is involved. Electro-thermal actuators, on the other hand, do not need a high voltage and still achieves comparable strain performance to electrostatic ones. Some other electrically driven soft actuators such as electrochemical actuators that uses ion migration for activation and electromagnetic actuators also do not require a high voltage. However, they often exhibit inferior output forces and are relatively complicated to fabricate, thus they will not be covered in this review. Chen et al. had proposed a review article that focuses on low-voltage soft actuators that are suitable for human–machine interaction [64]. Electro-thermal actuators are thermally activated actuators powered by Joule heating-heating by electricity. Their high output force lightweight nature and silent operation have made them a potentially attractive choice for artificial muscles that are applied in prosthetics. The twisted and coiled polymer (TCP) actuator is a newly emerged linear actuator that either contracts or extends upon activation. TCP is shaped like threads and therefore requires very little volume to be implemented, making compact designs possible. Compared with electrostatic actuators, TCP only requires a few or a few dozen volts to operate, making it a safer choice that can be operated without the need of a voltage amplifier. TCP has the ability for strain, force, and stiffness sensing via resistance monitoring [65,66,67]. Compared with a similar type of actuator, shape memory alloy coils, TCP exhibits the advantage of low hysteresis, no phase transition during actuation (so a more linear relationship between input and output), and significantly lower cost [68]. TCP’s working principle is the thermal expansion of the twisted fibers. The thermally expanded fiber would cause the untwisting of fibers and further the contraction or expansion of the whole actuator as shown in Figure 6. Contraction is achieved if twisting and coiling directions are the same, and expansion actuation is attained otherwise. Nevertheless, contraction exerts a much larger force than extension and thus is the pervasive actuation mode. TCPs have been reported to have specific energies in and peak specific works (work during contraction) that are magnitudes higher than electrostatic actuators. However, TCP actuation cycles are significantly longer than electrostatic actuators due to the time required for heating up and cooling down. Large actuations require heating over 100 °C, creating challenges for encapsulation materials and surroundings to ensure safety, especially if used in prosthetics. Furthermore, Joule-heating has a low energy efficiency, and all the supplied heat is wasted during the cooling down process, making the energy efficiency of TCP incomparable to electrostatic actuators. To date, TCPs are mostly applied in robotic arms and prosthetics.

Early versions of conductive twisted coil polymers were demonstrated by Lima et al. in 2012 [69]. The group made TCPs out of carbon nanotube yarns, and those actuators had been shown to lift loads 17,700 times their own weight over 3% strain under 18.3 V/cm and 20 Hz applied voltage. Despite the attractive actuation performance, CNT yarns are expensive for prototyping and production. To reduce the cost problem, Haines et al. proposed the use of nylon 6,6 and nylon 6 fishing lines to manufacture a cheaper (~$5/kg) and more powerful actuator [70]. The group proposed two coiling techniques: super-coiling (self-coiling) by continuous twisting and wrapping the fibers around a mandrel. Both techniques require annealing to stabilize the fibers into the coiled configuration. By heating super-coiled nylon 6,6 actuators from 20 °C to 120 °C, maximum strains of 21% at loading stress of 22 MPa and 9.3% at 50 MPa were achieved. Meanwhile, mandrel-coiled nylon 6 showed larger strain but less capability for loading stress. A maximum contraction of 49% contraction under 1 MPa loading stress was shown. Maximum specific energy of 2.48 kJ/kg and average specific power of 27.1 kW/kg during contraction were reported in general. Nevertheless, efficiency was around 1%, and specific power over a full activation cycle would be much lower than that during actuation due to extensive cooling time. The group showed that a self-coiled nylon 6,6 actuator could endure 1 million actuation cycles. Each cycle was powered by a voltage of 30 V/cm at 1 Hz that caused the actuator to lift 10 g (22 MPa) load at 20% duty cycle.

Scaling up force output could be achieved by weaving and braiding, as shown by Simeonov et al. [71]. The study focused on comparing 2D weaving, 2D braiding, and 3D braiding. Results showed that both weaving and braiding were good force amplification measures. 3D braiding was the best for force amplification and energy efficiency but had a lower bandwidth relative to 2D braiding. Braiding outperformed weaving in force amplification, bandwidth, and energy efficiency overall, but weaving may be essential for applications where flat actuators are desired over bundled ones. Self-sensing can be made by relating string length with string resistance as Bombara et al. showed that as the string elongated, resistance also increased as a result of a larger distance between adjacent coils [72]. Performance optimization through different plying patterns was studied by Cho et al. [73]. The researchers compared force and strain performances of different coiled structures made of eight silver-coated nylon 6,6 fibers. In the study, it was suggested that plying of TCA could form an anti-untwisting structure. Three structures: 2-4 (2 ply, 4 strings twisted together during twisting) TCA, 4-2 TCA, and 8-1 TCA were compared, and 2-4 TCA was reported as the optimal structure as it showed the largest strain under the same testing conditions. A 12.3 cm sample of 2-4 TCA reached a maximum strain of 17% when lifting 1000 g of load upon heating from 30 °C to 120 °C. As an application of their work, the group demonstrated the flexion and relaxation of a 3-joint biomimetic finger controlled by four serially connected 2-4 TCAs. The finger could grasp objects like a pen or USB stick. A materials measure to enhance the performance was done by Kim et al. [74]. The group presented a double-helix twisted and coiled actuator (DTCA) that merged non-conductive spandex TCP with conductive silver-coated nylon TCP to make the spandex conductive. The 8-strand spandex DTCA reached a maximum strain of 40% when lifting 1.9 N (194 g) while a 16-strand DTCA reached a 39% when lifting 3 N (306 g) of weight when both were Joule-heated from 20 °C to 110 °C.

Recently, Sun et al. proposed a free stroke TCP that, unlike other TCPs, could actuate without a preloading (pre-stretching) condition [75]. By wrapping the mandrel with a copper wire to act as a guiding groove and then wrapping a conductive nylon 6,6 following the groove, the group demonstrated a unique mandrel-coiling fabrication that allowed large uniform spacing between neighboring coils. The actuator reached 48% strain under lightly loaded conditions (0.03 g) and 55% strain under 10 g loading condition. The study also presented a programmable fully soft robotic arm composed of three types of actuators that were made by embedding the free stroke TCPs into soft elastomers. The arm was composed of an arm unit, a wrist unit, and a hand unit. The arm unit was embedded with three independently controlled actuators to allow bending motions in 3 directions. The wrist unit was built by a coil shaped TCP that could uncoil during actuation, thus creating a twisting motion. The robotic hand was built with a soft body embedded with TCP and a strain-restricting layer to perform uncurling motion during actuation.

Another application of TCP is artificial skin, as shown by Almubarak and Tandesse [76]. The skin was made by embedding a pair of 2-ply silver-coated nylon 6,6 TCP into a rectangular pad made of (140 mm * 60 mm) Ecoflex-30 silicone. During actuation, the contraction of TCP would cause the morphing of the whole structure. As a result, two types of morphed structures were reported: one being an undulating structure that had one or multiple bulges, and the other being a bending structure with a free end inclined at an angle. The activated patterns were shown to closely resemble the shapes of elephant trunks, caterpillars, and underwater knife-fishes, thus claiming that this artificial skin could be useful for biomimetic soft robots. Another biomimetic design of active skeletal joints was implemented by Wu et al. [77]. The group encapsulated three mandrel-coiled TCP into silicone sleeves and integrated them in an antagonistic fashion into a bendable skeletal structure. Actuation of each TCP would result in the bending in one direction, and the antagonistic alignment of actuators allowed for faster restoration. The TCP was made of coiled nylon 6 fishing line with resistive Ni wire wrapped around for heating. It achieved a maximum strain of 49% when powered by 10 V. A maximum bending angle of 20° for the joint was reached within a second. The design was further enhanced by fan cooling as shown by Wu and Wang [78]. In the field of locomotive soft robots, TCP had been applied to build an inchworm-inspired robot by Yang et al. [79]. The body was built of silicone with two directional frictional feet attached at the ends. A supercoiled polymer actuator was attached to the ends and upon contraction, the body would bend. A bending angle of 16.5° and a crawling speed of 0.245 mm/s was reached for an 11 cm long robot. Other applications of TCP include haptic feedback [80], variable stiffness pneunet muscle [81], and soft pump [82].

To the best of our knowledge, there has been no efficacious way to merge a cooling system with TCP to give it a faster deactivation and thus higher bandwidth. Although passive cooling methods such as immersing in water or helium [70], and active fan cooling [68,78] have been used, problems like soaking or addition of bulky rigid parts can be an issue to many applications. Therefore, future research can be directed towards better cooling methods such as the use of water-resistant polymers, solid coolant, or highly thermal conductive polymer threads, but practically these actuators may be better used in applications with infrequent actuation and integrated with clutch mechanisms to reduce the need for active power to hold different positions. Another direction is to use the TCPs to actuate larger lightweight, 3D printed actuators with specially designed topology like the ones presented by Goswami et al. [83]. Their design used a motor-tendon to actuate, but in the future, similar design using TCP actuation can be made.

### 5.2. Phase-Changing Actuator

The phase-changing actuator is another type of thermally activated actuator. The volumetric expansion of materials is the only actuation scheme, and it relies on the increasing air pressure of internal cavities as a result of evaporation or melting of encapsulated phase changing materials (PCM). Due to the relatively smaller amount of research in solid to liquid actuators and the undesired stiffness of the solid phase, this review only covers PCM actuators that go from the liquid to gas phase. The PCM actuators are heat-actuated, hence any heating method can drive the actuator. Electrically actuated and controlled PCM actuators can be achieved by embedding heating wires [84,85,86,87]. Other methods include microwave [88], near-infrared actuation [89], and induction heating [90,91,92]. Phase-changing actuators are unique due to their volumetric expansion mode of actuation. They have excellent performances in strain and exerted force while requiring actuation voltages below 50 V. Furthermore, phase-changing materials usually have energy densities well above electrostatic actuators [93]. These merits make phase-changing actuators a potential alternative to elastic inflatable actuators. Nonetheless, there are several major drawbacks. First, actuation cycles are extremely slow (above 150 s typical, 20 s lowest [88,94] due to the time required for heating up and cooling down. For full actuation, heat must be transferred through the whole actuator, but the low thermal conductivity of materials such as silicone would hinder such processes. Second, lifetime is very limited (few dozens to few hundreds of actuation cycles) due to the escape of PCM vapor and elastomer degradation at high temperatures. Finally, reported energy efficiency is only 0.2% [86], which is orders lower than electrostatic actuators. Ethanol [86,95,96] and Novec-7000 [92,97,98] are the most commonly used materials for PCM, although other liquids like water [94] and FC-72 [99] have also been presented. Three models of encapsulation are present: 1. an elastomeric matrix with embedded/mixed PCMs (elastomer composite) [85,86,88] 2. elastomeric pouch containing PCMs [89,92,100] 3. inextensible pouch contained with PCMs [91,97].

**Figure 7:**
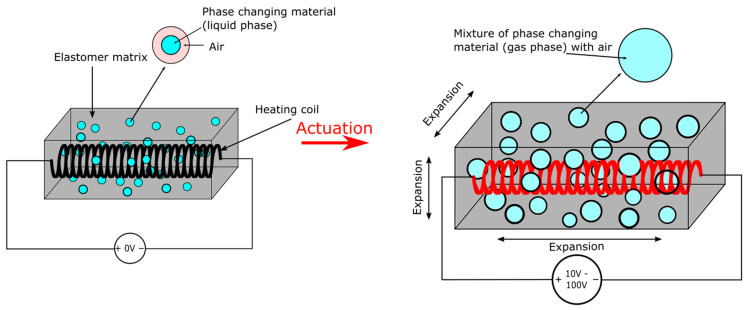
The actuation scheme of an elastomer composite actuator. Electric actuation requires a voltage typically between 10 V to 100 V to power a resistive heater that is either embedded or external to the actuator. The heating will cause myriads of embedded bubbles to expand due to the increased vapor pressor (from the vaporization of the phase changing material), which overall causes the volumetric expansion of the whole actuator.

Elastomer composite actuators, as shown in Figure 7, have been applied to make soft robotic grippers [86], locomotive robots [94], and artificial muscles [84,86]. The first elastomer composite actuator was presented by Miriyev et al. in 2017 as a form of soft actuator that could expand upon Joule heating [86]. The actuator was prepared by mixing 20% by volume of ethanol with Ecoflex 00-50 silicone to form an elastomeric composite. A coil-shaped Ni-Cr heating wire was embedded into the actuator before curing. The resulting actuator could expand volumetrically by 915% and linearly to 100% when heated to 90 °C by a 15 V, 1 A power supply. Under the same power condition, a 6 g actuator was presented to exert a unidirectional force of 60 N for 30 repeated cycles over a time interval of 5000 s (150 s/cycle), and a 2 g sample could output 120 N of maximum force (1.3 MPa of stress) which was 6000 times the actuator’s weight. By covering the soft actuator with a braided mesh sleeve, the group made a McKibben muscle weighing 13 g and lifted 1 kg of weight with 25% strain under 30 V, 1.5 A of power. Antagonistic pairs of McKibben muscles were also attached to a skeletal arm to create larger displacement and faster actuation. By adding a strain-restricting layer to a rectangular actuator, both Miriyev et al. and Li et al. [85,86] showed bending locomotive soft robots that mimicked the crawling motion of worms. The two groups, although in different styles, also showed elastomer composite soft grippers capable of delicate tasks like picking up an egg and strawberry. However, low actuation speed and high temperatures made this type of actuator not as suitable for the above applications as electrostatic or TCP actuators.

Additive manufacturing via 3D printing of actuator fibers using a specially made silicone composite 3D printer was presented by Miriyev et al. in 2019 [101].

Utilizing the unique volumetric expansion characteristic, Decroly et al. proposed voxel modules that could perform actuation in six degrees of freedom including bending, twisting, shear, compression, and extension [98]. The voxel was made by a silicone (Ecoflex 00-30) box with embedded strain-restricting paper kirigami structure that constrained expansions in certain directions, thus forcing the voxel to expand in a designed anisotropic fashion.

To improve actuator speed of operation, Miriyev et al. suggested to keep the actuator warm when idle [95]. Doing so eliminates the time required to reach the threshold temperature of evaporation.

A materials approach to solving the same problem by embedding thermally conductive diamond nanoparticle-based filler to boost the heat conductivity of the silicone was presented by Xia et al. [96]. The operation duration of a silicone-ethanol matrix actuator dropped significantly from 402 s to 125 s as thermal conductivity increased from 0.190 W/mK to 0.248 W/mK after the addition of 20 wt.% of the filler. This method was also demonstrated to increase lifecycles from 40 cycles of 0 wt.% specimen to near 120 cycles of 20 wt.% specimen because of better uniform heating which reduced thermal degradation around the heating element.

An all-soft actuator was achieved by replacing heating wires with expanded intercalated graphite (EIG) silicone conductive composite [84]. The EIG-silicone composite was soft, heating-conducting, electrical-conducting, and plasticized into arbitrary shapes. The study showed that EIG-composite with certain shapes allowed better uniform heating, and thus increased the heating process. Furthermore, this material also made 3D printing of Joule-heated actuators possible.

Limited lifetime is a result of two factors: PCM evaporation/escape, and degradation of the elastomer from prolonged heating. PCM escape can be partially addressed by a method called rejuvenation, as proposed by Miriyev et al. [102]. The method showed that by simple immersion of degraded actuator into PCM, the actuator would reabsorb the liquid until saturation at 100% of its pristine amount of PCM. However, the duration of the process is dependent on the size of the actuator and often takes a significant amount of time (45 h for a 1 cm radius cylinder and 101 h for a 1.5 cm radius cylinder) to reach 99% of pristine PCM volume.

Another approach to reducing PCM escape is to use elastomers that are less permeable to certain PCMs. For example, Noguchi and Tsumori [88] built and compared four actuators, one with silicone (Ecoflex 00-10) and water, one with silicone and Novec 7000, and the other two using the same PCM but urethane rubber elastomer since urethane exhibit lower gas permittivity than silicone. After 3 h of exposure to air, the silicone + Novec 7000 sample had no remaining gas contained, while the silicone + water still preserved some vapor after 5 h. Both urethane samples contained vapor after 5 h. The results did indicate that materials with lower permeability to PCMs would better preserve the PCMs.

A study on the actuation strain and speed of different materials was presented by Decroly et al. [98]. The study compared Novec-7000, acetone, methanol, ethanol, isopropanol, and acetonitrile as materials for PCM; Ecoflex 00-30, Ecoflex 00-50, and Dragonskin 10 for silicone elastomeric matrix. The results showed that Novec-7000 performed the best in terms of maximum strain and strain rate, followed by ethanol-based PCMs (ethanol, methanol, isopropanol). A Novec sample achieved a strain of 71% and strain rate of 0.2%. Novec-7000 had the lowest boiling point and least latent heat, but there was no overall trend between them and maximum strain or strain rate. Concerning the silicone matrix, Ecoflex 00-30 showed the highest strain by virtue of its lowest modulus and tensile strength, while Dragonskin 10 showed the lowest strain for the opposite reason.

**Figure 8:**
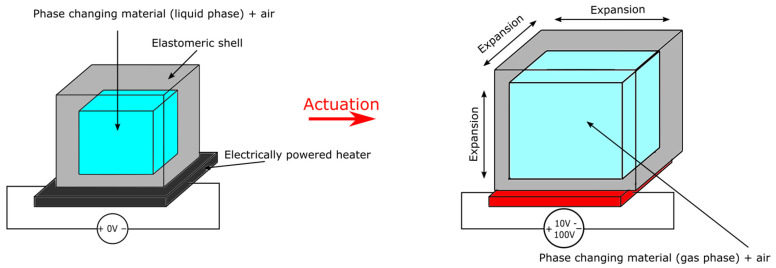
The actuation scheme of an elastomeric pouch actuator. The external resistive heater is required with a powering voltage typically between 10 V–100 V. Heating above the phase changing material’s boiling point will cause vaporization. The increased gas pressure will cause volumetric expansion of the actuator.

Different from elastomer composite which has myriads of small bubbles embedded into an elastomeric matrix, the elastomer pouch actuator is an elastomeric pouch with PCM filled within it in a large chamber as shown in Figure 8. Elastomer pouches are commonly used as an alternative to traditional pneunet actuators that require external compressors. For instance, Chellaton et al. and Matsuoka et al. implemented bending actuators that closely mimicked a typical bending pneunet [99,103]. The actuators used the thermal expansion during PCM phase change instead of an external pressure source. The actuator proposed by Chellatoan et al. was made of a steel wool yarn conductor being inserted into a silicone pneunet filled with ethanol. Maximum bending was achieved within 40 s under 30 W (12 V, 2.5 A) of power. The actuator proposed by Matsuoka et al. showed a larger bending motion that closely replicated an identically shaped pneumatic actuator when heated by a heating rod driven by 60 V applied voltage. Applying elastomer pouch to the field of soft robotics, Li et al. made a soft gripper that could grab small objects [94] and rolling locomotive robots by attaching multiple actuators onto a rigid wheel. Another rolling soft robot was demonstrated by Nishikawa and Matsumoto [92]. The cubic robot had four chambers to hold pouch actuators made of natural rubber and Novec 7000. Upon contact with a heating surface, one actuator would expand, which tilted and eventually flipped the cube. The continuous tilting and flipping created locomotion. A large expansion micro elastic pouch was presented by Hirai et al. [100]. The 5 mm latex + Novec-7000 actuator could expand to 84 times its inactivated volume upon heating to 60 °C. It was demonstrated to actuate the linear motion of a piston with the 20 N of force after 250 s of heating.

**Figure 9:**
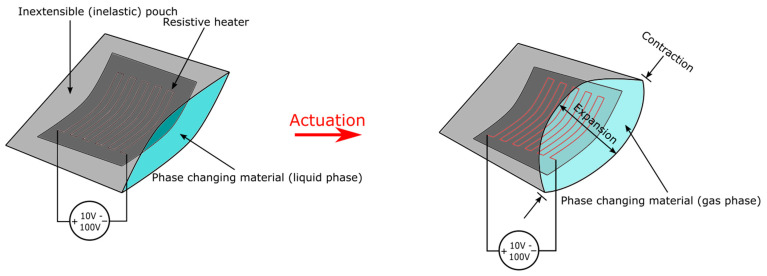
The actuation scheme of the STAT actuator. A resistive heater is embedded within the actuator, and power of 6 W is required to heat up the actuator. Since the pouch is inelastic, vaporization of the changing material will bulge the pouch.

Using an inextensible shell to encapsulate PCMs, inelastic pouches (Figure 9) are actuators that stay flat and compliant when inactivated, bulged and pressurized when activated. It is commonly used for active textiles. For instance, Sanchez et al. proposed the smart thermally actuating textiles (STAT) using PCMs [87]. The textile was fabricated using thermal bonding techniques and exhibited a laminated pouch design that enabled Joule heating and pressure-sensing of the textile. Due to the requirement of close contact with humans, Novec 7000 was chosen as the PCM due to its low boiling point. The actuator was capable of producing 75 kPa pressure after 15 s of heating by 6 W of power but required 140 s to cool down. A closed-loop pressure controller was implemented based on pressure-sensing. A wide range of applications for STAT includes modular mechanotheraputic wearables, responsive cushions, artificial muscles (in ways similar to Peano-HASEL), and dynamic fashion. A simpler design, called liquid pouch motors, was presented by Narumi et al. [97]. The group showed serially connected rectangular motors that were similar to an electrothermally driven Peano-HASEL. However, these motors were much lighter in weight while capable of performing larger actuation strain. Nevertheless, actuation time was notably longer than Peano-HASELs. Compared to STAT, liquid pouch motors lack the presence of pressure sensors but can be massively fabricated in a simpler roll-to-roll fashion. Using that technique, 2000 motors were prepared for the actuation of decorative butterflies and installed on a dome architecture. The simplicity of the actuator’s structure also allowed elaborated design using CNC heat-drawing just like thermoplastic HASELs. Other applications of inelastic pouch include haptic displays like the HaPouch presented by Uramune et al. [104], and wirelessly activated pneumatic bellows-style actuators that can be applied to organ operation and unlocking of channels in the future [91].

Some of the actuators discussed above are driven by induction or wireless heating, such as the rolling robot made by Nishikawa and Matsumoto [92] and the bellow-style actuator made by Boyvat et al. [91]. A simple redesign can convert them into Joule-heated actuators. Despite some of their merits, all phase changing actuators exhibit the problems of slow actuation and limited lifetime. Although several measures to address these issues are known, the performance is still not acceptable for many applications. If the actuation times can be reduced to a few seconds while extending the lifetime to over 10,000 cycles in the future, this type of actuator could show much greater potential.

There are also some other electro-thermal actuators that uses a different actuation principle than the ones discussed above. For instance, the reprogrammable ss-LCE (disulfide liquid crystal elastomers) actuators proposed by Wang et al. contained a resistive heating wire for efficient activation, and the actuator exhibited different modes of morphing after being reprogrammed using heat and tension [105]. Actuation of this actuator was achieved by the state transition of the liquid crystal polymers upon heating/cooling. Another example is Taccola et al.’s electrically controllable hydromorphic soft actuator that bends upon Joule heating. The design comprised an active thin (hundreds of nm) poly(3,4-ethylenedioxythiophene):polystyrene sulfonate (PEDOT:PSS) film and a thicker (100 + um) train-constraining PDMS film [106]. The PEDOT:PSS is a special material that desorbs H_2_O when heated and reabsorbs H_2_O in ambient air when cooled down. This trait gives the material the ability to contract upon heating and expand upon cooling. Due to its thinness, the actuator was responsive to 5 Hz driving signal, which was higher than most other electro-thermal actuators. Other forms of electro-thermal actuators include bilayer electro-thermal actuators that uses different of coefficient of thermal expansion [6] to curve/curl and shape-memory alloy/polymers. However, they will not be discussed in detail in this review, and a detailed review on them was written by Tian et al. [107]. 

**Figure 10:**
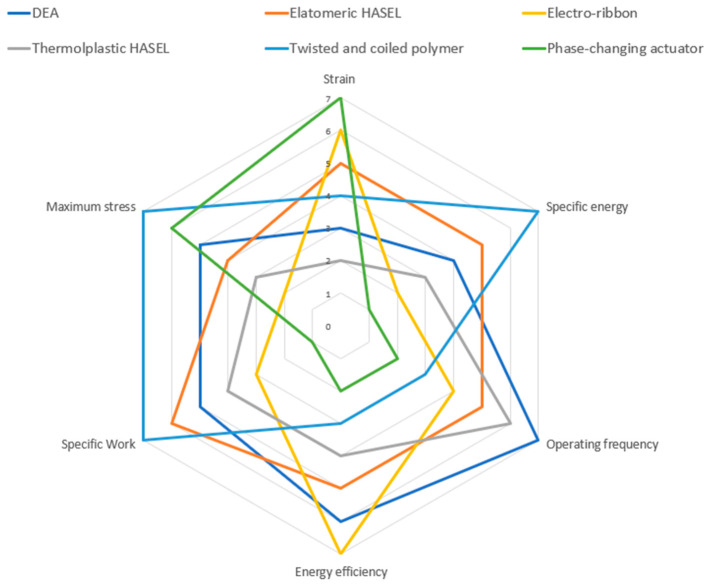
Qualitative comparison of the key parameters of each type of actuator. The rating number (0–6) was assigned based on the ranking of the higher end of the typical performance (shown in Table 2) of each type of actuator. A rating of 2 means the poorest performing type of actuator, while a rating of 7 means the best. A rating of 1 means that the we could not find any reported value of the parameter for that type of actuator.

Table 1 compares the qualitative characteristics of all types of actuators disccused in this review. A quantitative comparison of the performances of each type of actuator is shown in Table 2. Figure 10 shows a qualitative comparison in performances of each type of actuator. 

## 6. Electrically Driven Soft Pumps

In the field of soft robotics, pneumatic and hydraulic actuation schemes still remain the most popular ones. Nevertheless, pumping air or liquid into the actuators requires rigid and bulky pumps traditionally, making the soft machines not entirely soft. Furthermore, traditional pumps are power-consuming, rigid, heavy in weight, and loud, making them a less desirable choice. In recent years, numerous soft pumps have emerged in tandem with the development of soft robotics, and a selection of different pumping methodologies, applications, and relative performance is reviewed here.

## 7. Electrohydrodynamic Pumps

**Figure 11:**
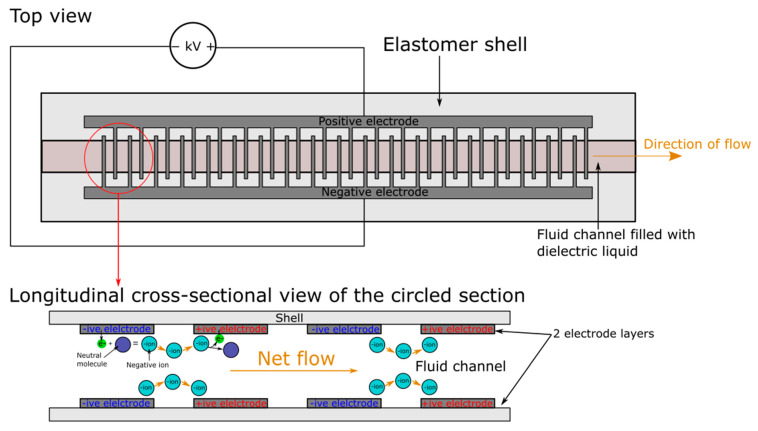
The structure of a basic soft stretchable pump. Illustration inspired by Cacucciolo et al. [111]. There are two comb electrode pairs, one located at the top layer of the shell and the other at the bottom layer. Both electrode pairs are responsible for ionizing liquid molecules and moving the ionized molecules from left to right using electrophoresis.

Electrohydrodynamic (EHD) pumping, which describes the flow of dielectric or ionized fluid under an applied electric field, has enabled direct fluid pumping without the use of any moving mechanical parts. EHD has three types of working modes: ionic-drag, conduction, and induction [109,110]. With respect to current application in soft pumps, ionic-drag EHD is the dominant type due to its potentially high flow rate and relatively simpler design. A well-known soft EHD pump is the soft stretchable pump presented by Cacucciolo et al. in 2019 [111] (Figure 11). The pump had dimensions of 75 mm * 19 mm * 1.5 mm and weighed 1 g. PDMS was used for the bulk material and comb-patterned electrodes made of silver ink or carbon-loaded PDMS were embedded into the bulk elastomer. Fluorinert FC-40 was used as the dielectric pumping liquid. When enough voltage was applied, the energies within the electrons of the cathode material would rise above the barrier energy and directly tunnel into the dielectric liquid (field emission), forming anions with molecules in the liquid. The anions then accelerate toward the nearest anode where they discharge, dragging nearby neutral fluid molecules along (electrophoretic flow) [109]. In terms of performance, pumps made of both electrodes could move liquid at 50% strain, however, stretching the pumps would cause a degradation of fluid pressure. The activation voltage of the carbon pump was around 2.5 kV, half of the minimum voltage required by the silver pump, meaning the carbon pump could react faster and required lower applied voltage. When actuated using a 4.5 kV square wave, the carbon pump was demonstrated to reach maximum pressure within 0.4 s and drop back to 0 kPa within 0.14 s, making it capable of fully reacting to signals above 2 Hz. However, the maximum applicable voltage to the carbon pump is also around half of the silver pump (5.6 kV versus 10 kV), making the carbon pump only capable of creating 7 kPa of maximum pressure at 5.6 kV as compared with the 14 kPa generated by the silver pump at 10 kV. Moreover, the carbon pump drew much more current than the silver pump and exhibited a lifetime of only 15 min, which was significantly lower than the hours-long operation of the silver pump. Furthermore, the flow direction of the silver pump could be altered by reversing the polarity of the applied voltage. The maximum reported flow rate for the stretchable pumps was 6 mL/min. In terms of applications, the pump was shown to power a soft textile glove for on-body temperature regulation, a bending soft hydraulic actuator, and a Mckibben muscle.

**Figure 12:**
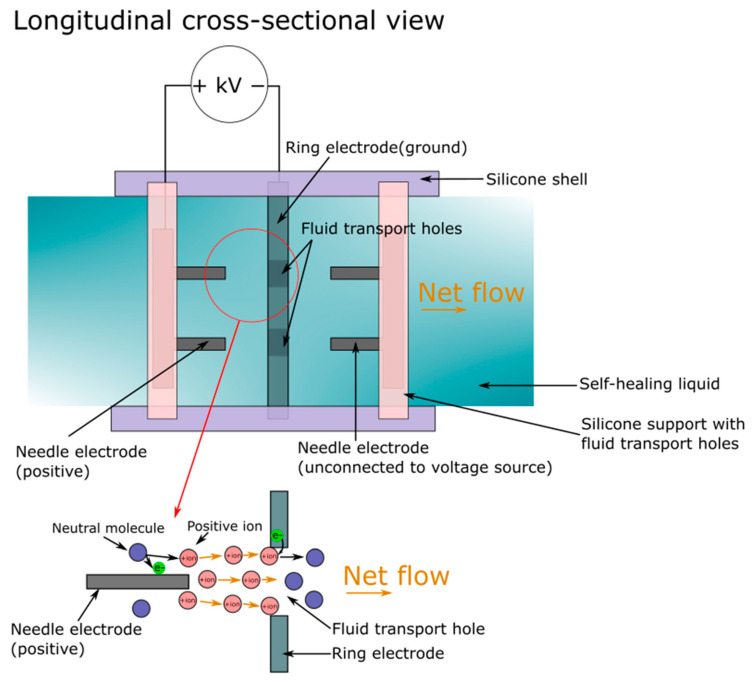
The structure of a cylindrical self-healing soft pump. Illustration inspired by Tang et al. [112]. There are two electrode pairs (pair1: left needle electrode + ring electrode, pair2: right needle electrode + ring electrode), and only one pair is powered at a time. Flow direction can be switched by disconnecting the left needle electrode and connecting the right needle electrode to the voltage source.

A soft stretchable pump could be revolutionary as it opens the door for fully soft static pumps, but the current performance is not adequate for many heavy-duty applications. A more recent self-healing soft pump presented by Tang et al. showed significant performance improvement [112] To enhance the performance of ionic-drag EHD, the group used a positive ion migration principle to drive the pump. In contrast to the 2D structure of the previously discussed soft stretchable pump, the pump described by Tang et al. consisted of a 3D cylindrical structure with two electrode pairs implanted into the soft shell. Positive electrodes were made of numerous needle electrodes attached to a base electrode, and grounding electrodes were made of a base electrode with the corresponding number of holes on the base (Figure 12). The combination of two positive electrodes with one grounding electrode would form two electrode pairs that allowed bidirectional pumping by switching between the two pairs. Self-healing was achieved via the use of dibutyl sebacate-tung oil solution as the pumping liquid. The solution fully solidified upon contact with oxygen at room temperature after 1 day of exposure. A maximum pressure of 9.2 kPa and flow rate of 423 mL/min was reported for one needle-hole pair. A maximum pressure of 60 kPa, flow rate of 521 mL mL/min, specific pressure of 3067 kPa/kg, and specific flow rate of 141,000 mL/(min kg) was shown overall, surpassing the values reported for the soft stretchable pumps. A response (peak) time of 0.45 s was stated, similar to the stretchable pumps, and direction switching could be made within 0.58 s. Scaling can be done by serially connecting multiple electrode pairs or adding more needles to electrode pairs. The lifetime of this pump is shown to be over 4 h of continuous operation at a 15 kV (2 h at the positive voltage and the other 2 h at negative), the generated pressure gradually dropped for the first hour and stabilized for the remaining hour (for both voltage polarities). The silver stretchable pump also exhibited a similar degradation pattern during lifetime testing. Tang et al. suggested that this pattern was the result of electrode passivation and stabilization [112]. In terms of application, the pump was applied to actuate a soft robotic fish and a rigid locomotive robot. Furthermore, the shape of the pump can be customized to fit different designs, and fabrication using 3D printed thermoplastic elastomers was demonstrated in the study.

## 8. Electro-Pneumatic Pump

**Figure 13:**
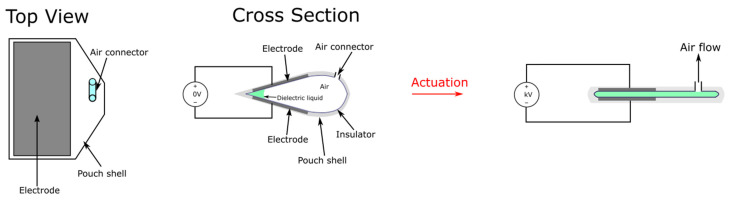
The structure of an electro-pneumatic pump. Illustration inspired by Diteesawat et al. [114]. The electrostatic force generated between the electrodes will be amplified through the dielectric liquid bead, causing a zipping motion of the pouch. During zipping, air will be squeezed out from the air connector.

Although the EHD pumps have great potential, there are also other soft pumps reported in the literature that use electrically driven soft robotic actuators to provide the energy for mechanical soft pumps. The first of these types is the electro-pneumatic pump (EPP) as shown in Figure 13. By using an inextensible thermoplastic pouch, structured like a Peano-HASEL, but with only a bead of dielectric liquid, Diteesawat et al. constructed a 5.3 g, 1.1 mm thick soft EPP based on electrostatic zipping amplified via dielectric liquid [113]. By punching a hole at a side of a region without electrode coverage and connecting the hole to a pneumatic tube though a connector, an inflated pouch can transfer air through the tube upon electrostatic zipping of the active region when powered by high voltage in the kilovolt range. The pump presented in the research was constructed of thermally bonded low-density polyethylene for the pouch body, copper tape for electrodes, PVC insulating tapes to cover the outer surfaces of electrodes, and a PVC backing layer in between electrodes and pouch walls to provide stiffness. By connecting the pump to a bubble artificial muscle [114,115], the group was able to make a linear artificial muscle system and measure the performance of the pump. A maximum contraction of 31.48% was achieved under 26.5 g loading and 17 mL injected air at 8 kV driving voltage. A maximum pressure of 2.34 kPa, specific pressure of 472 kPa/kg, bandwidth over 10 Hz (1 Hz for close to 100% contraction), and energy efficiency of 46.5% was achieved. A maximum flow rate of 161 mL/min and specific flow rate of 20.5 L/(min kg) were also reported, surpassing that of soft stretchable pumps. As an application, the group attached an artificial muscle system constructed by 2 EEPs with 3 BAMs to a plastic skeletal arm to actuate bending motion of the arm. The linear contraction of the 11 cm long muscle caused a large bending of the arm as the tip of the arm moved 23 cm when the pump was activated at 10 kV.

In comparison to the EHD pumps, this pump can pump air instead of liquid, is more lightweight, and has a better lifetime of >8000 actuation cycles. Nevertheless, activation will cause large mechanical motion which may be undesirable, meanwhile, the EHD pumps operate with no mechanical movement and can produce continuous rather than pulsed flow.

## 9. DEA Pump

**Figure 14:**
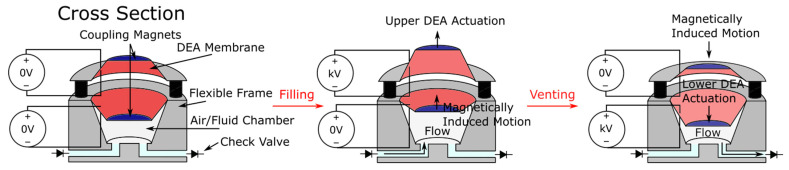
The structure of a magnetically coupled DEA pump. Illustration inspired by Cao et al. [116]. Since the two DEA membranes are magnetically coupled, actuating one will induce a motion of the other. By alternatively actuating the top and bottom DEAs, the air/fluidic chamber will experience alternative negative and positive pressures, which results in the net flow of air or fluid from the left inlet to the right outlet.

DEAs have been integrated into soft pumps in various applications, and of the reported versions, the magnetically coupled DEA pump (shown in Figure 14) proposed by Cao et al. demonstrated excellent performance in terms of flow rate and pressure [116]. The pump was constructed of two magnetically coupled DEA membranes which were attached to a rigid frame. Two check valves were connected to the frame. The top membrane was separated at a distance from the lower membrane due to magnet repulsion, and the lower membrane was sealed to the rigid frame, forming an air chamber. The first half of actuation would cause both membranes to displace upwards, drawing air into the chamber. The second half of actuation would displace both membranes downwards below the un-activated position, forcing air out of the chamber. A circular pump of 3 mm diameter could produce a maximum air pressure of 3.02 kPa and a flow rate of 0.92 L/min when operated at a resonant frequency of 77 Hz when using 4.5 kV applied voltage.

## 10. TCP Pump

**Figure 15:**
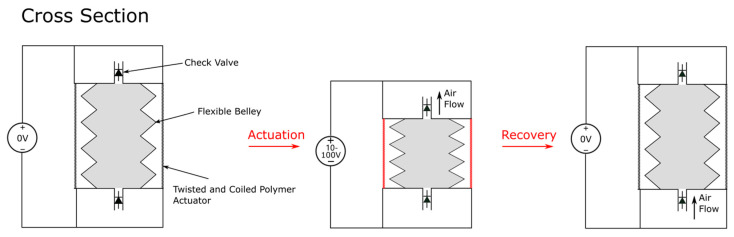
The structure of a TCP pump. Illustration inspired by Tse et al. [82]. Joule heating the TCPs using an electrical power source will cause the TCPs to contract, which compresses the flexible bellow and pushes air out through the top check valve. During the cooling down process, the bellow will recover to its uncompressed state due to elasticity and air will refill the bellow through the bottom check valve.

A low voltage TCP actuated soft pump is presented by Tse et al. [82] as shown in Figure 15. The pump was constructed with a soft bellow membrane and two rigid fixtures at the ends of the membrane to attach SCPs. Actuation was made by joule heating of the SCP using voltages in the range of 12 V–24 V. The contraction of SCP would displace the bellow, then the cooling down of SCP would allow the bellow to restore its original position, forming a pumping cycle. A 6 cm high, 5 cm diameter pump weighing 50 g could produce a maximum pressure of 2.63 kPa, flow rate of 54 mL/min, specific pressure of 52.6 kPa/kg, and specific flow rate of 1.08 L/(min kg). The time required for a complete actuation cycle was 10 s. The performance in terms of pressure, flow rate, and bandwidth are not as good compared to previously reported pumps, but it is the only soft pump in this review that can be activated using a very low voltage.

## 11. Summary

In conclusion, this review article is aimed to provide an overview and comparison of many mainstream, state-of-the-art electrically driven soft actuators. Furthermore, this review also serves to provide a start-up guide for the understanding, selection, and further researching of many state-of-the-art electrically driven soft actuators and pumps for students (and scholars) who are new to the field of soft robotics. This review is focused on characteristic description, parameter comparison, and application comparison of each type of actuator. Compared with many hydraulic, pneumatic, and other soft actuators, electrically driven soft actuators have the benefits of theoretically higher energy efficiency, scalable manufacturing, and improved performance with size reduction, the ability to self-sense, portability, and easier integration with digital systems. The scope of this article includes dielectric elastomer actuator (DEA), electro-hydraulic soft actuator (mainly HASEL actuator), electro-ribbon actuator, twisted and coiled polymer actuator, phase-changing (liquid to gas) actuator, soft electrohydrodynamic pumps, soft electro-pneumatic pumps, and soft pumps made of soft actuators. For all the actuators listed here, the primary challenges to wide-scale implementation are the requirements of high voltage for DEAs and HASELs which have a number of safety and reliability issues, and the extreme inefficiency of the TCP type artificial muscles. There may yet be solutions for these issues in the future, for example as the thickness of polymer and electrode layers is reduced, the voltages required to achieve the high fields needed for DEAs to operate are drastically reduced. Thermoplastic elastomers, which can be cast from solvent or thermally drawn are not as widely used yet in these DEA and HASEL applications and could possibly replace or improve on silicone rubbers for future implementations. Self-healing polymers, fluids, and electrodes could become more widely used as a mechanism to enhance the lifetimes of high voltage actuators, and more efforts could be expended on standardization of power systems and safety integration so that more accessible systems could be available to researchers with more limited budgets. To date, there is as yet no equivalent to the electromagnetic motor in the soft robotics field—a single actuator that is well known and acceptable to a broad range of applications. There have been notable strides towards something like this however and the state of the art has improved dramatically over the last decade and new manufacturing approaches and materials combined with application driven development should put these materials closer to the center of the burgeoning soft robotics field.

## Figures and Tables

**Table 1:** Qualitative comparison between different types of actuators.

Type of Actuator	Working Principle	Best Applications	Merits	Drawbacks
DEA	Maxwell stress created by electrostatic force	High-frequency artificial muscles	High bandwidth Self-sensing through capacitance monitoring	Requires a high voltage Susceptible to dielectric breakdown
Elastomeric HASEL	Maxwell stress created by electrostatic force	Artificial muscle	Self-healing Self-sensing through capacitance monitoring	Requires a high voltage
Thermoplastic HASEL	Electrostatic zipping amplified by a dielectric liquid	Artificial muscle Mass-produced actuators	Simple and cheap fabrication with complex designs Self-sensing through capacitance monitoring	Requires a high voltage Dielectric fluid may leak
Electro-ribbon	Electrostatic zipping amplified by a small drop of dielectric liquid	Active Origami designs Lightweight, large stroke contractive actuators	Easy fabrication Light-weight Self-sensing	Requires a high voltage Slow actuation Liquid leakage
Twisted and coiled polymer	Contraction or expansion upon Joule heating	Linear contracting artificial muscle Small-volume applications Lightweight applications	High specific energy Low voltage actuation Light-weight High strain	Long actuation period Low energy efficiency
Elastomer composite and elastomeric pouch phase changing actuators	Volumetric expansion due to the vaporization of a phase changing material	Artificial muscle that requires a large force Voxel actuators Soft robotic muscle	Very large strain High specific energy Low voltage actuation Wireless actuation	Long actuation cycle Limited lifetime Low energy efficiency

**Table 2:** Quantitative comparison of actuator key parameters. The values marked typical are obtained based on the authors’ overall understanding of the technology.

Type of Actuator	Linear Strain	Operating Frequency	Specific Energy	Specific Work	Maximum Stress	Energy Efficiency
DEA	3–30% typical (Max at 215% [14])	0–550 Hz typical (Max > 1000 Hz [17,18])	<20 J/kg typical (Max at 1150 J/kg [108])	100–200 W/kg typical (Max at 1000 W/kg [18])	<1 MPa typical (Max at 7.2 MPa [14]))	1–30% typical (Max at 90% [14])
Elastomeric HASEL	30–50% typical (Max at 124% [42])	0–20 Hz typical	70 J/kg typical [42]	Average specific work of 337 W/kg typical (Peak power at 614 W/kg [42])	0.002–0.3 MPa typical [42]	21% typical (Data from donut HASEL [42])
Thermoplastic HASEL	10–25% typical (Max 118% [51])	0–25 Hz typical (Max at 50 Hz [43])	5–12 J/kg typical (Max at 12 J/kg [44,51])	Average specific work of 100–180 W/kg typical (Peak power at 365 W/kg [49])	0.03–0.2 MPa typical (Max at 0.21 MPa [43,44])	13.6–19% Typical [51]
Electro-ribbon	99.84% [59]	0.125 Hz for full actuation [59]	6.88 J/kg [59]	51.45 W Average (Peak power at 104 W/kg [59])	0.048 MPa [59]	70% [59]
Twisted and coiled polymer	20–50% typical (Max at 55% [75] from a free-stroke TCP)	<1 Hz typical (Max at 20 Hz, [70], 3% actuation at this frequency)	1000–2000 J/kg typical (Max at 2480 J/kg [70])	1000–27,900 W/kg typical (27,900 W/kg [69]. 27,100 W/kg [70])	5–85 MPa typical (Max at 105 MPa [69], tensile contraction during this actuation was above 1%)	<1% typical (Max at 1.32% [70])
Phase-changing actuator	140% (Theoretical maximum at 140% (with no applied force) linear strain [86])	<0.007 Hz typical (Actuation cycle of over 150 s [86]. The lowest reported actuation cycle is 20 s, which converts to 0.05 Hz. [88,94])	-	-	0.05–1.3 MPa typical (Max at 1.3 MPa [86])	0.2% (Calculated from the expansion process of a 20 vol% ethanol + PDMS matrix elastomer composite actuator [86].

## Data Availability

Original data is the property of the primary articles referenced within.
